# Interventions integrating health and academic interventions to prevent substance use and violence: a systematic review and synthesis of process evaluations

**DOI:** 10.1186/s13643-018-0886-3

**Published:** 2018-12-06

**Authors:** Tara Tancred, Sara Paparini, G. J. Melendez-Torres, Adam Fletcher, James Thomas, Rona Campbell, Chris Bonell

**Affiliations:** 1 Department of Public Health, Environments and Society, London School of Hygiene and Tropical Medicine, 15–17 Tavistock Place, London, WC1H 9SH UK; 2 Division of Health Sciences, Warwick Medical School, University of Warwick, Coventry, CV4 7AL UK; 3 Centre for the Development and Evaluation of Complex Interventions for Public Health Improvement (DECIPHer), School of Social Sciences, Cardiff University, Cardiff, CF10 3WT UK; 4 EPPI-Centre, Department of Social Science, UCL, London, WC1H ONR UK; 5 Department of Population Health Sciences, University of Bristol, 39 Whatley Road, Bristol, BS8 2PS UK

**Keywords:** Process evaluation, Systematic literature review, Health education, Tobacco use, Substance use, Violence

## Abstract

**Background:**

Within increasingly constrained school timetables, interventions that integrate academic and health education to reduce substance use and violence may hold promise as a category of intervention that can positively affect both academic and health outcomes. There are no current systematic reviews exploring the effectiveness of such interventions or factors that affect their implementation.

**Methods:**

A total of 19 bibliographic databases and 32 websites were searched. References were also extracted from the reference lists of included studies, and experts and authors were contacted to identify relevant studies.

We included reports with no restrictions on language or date. References were screened on title/abstract and those not thus excluded were screened on full report. Data extraction and appraisal followed the Critical Appraisal Skills Programme, Evidence for Policy and Practice Information and Co-ordinating Centre and Cochrane tools. Extracted process data were qualitatively meta-synthesised for common themes.

**Results:**

Seventy-eight thousand four hundred fifty-one unique references were identified, and 62 reports were included. A total of 16 reports (reporting on 15 studies of 12 interventions) evaluated process. Key facilitators of integrated academic and health curricula were supportive senior management and alignment of the intervention with school ethos; a positive teaching environment, including positive perceptions around the ability to be flexible in the adaptation and delivery of integrated academic and health curricula; positive pre-existing student and teacher attitudes towards intervention content; and parental support of interventions, largely through reinforcement of messaging at home. Important barriers were over-burdened teachers, with little time to learn and implement integrated curricula.

**Conclusion:**

Several useful facilitating and inhibiting factors linked to the implementation of interventions that integrate academic and health education for reduced substance use and/or violence were identified, providing tentative but insightful evidence of context-specific issues that may impact intervention success. However, overall, there is still a considerable gap in our understanding of how to achieve the successful implementation of these interventions.

**Electronic supplementary material:**

The online version of this article (10.1186/s13643-018-0886-3) contains supplementary material, which is available to authorized users.

## Background

Schools have long played a role in promoting health among students [1–5]. However, schools in many countries now dedicate less curriculum time to health-related programming due to increasing pressures to meet academic performance standards, which place constraints on school schedules [6–8]. One way to maintain health-promoting programmes that is receiving increasing attention is through the integration of health and academic education curricula [9, 10]. To assess our current understanding of these curricula, we carried out a systematic review of such interventions aiming to prevent tobacco, drug or alcohol use (henceforth referred to as ‘substance use’) and/or violence. To our knowledge, this review is the first of its kind. As part of the systematic review, theories of change, process and outcome evaluations were synthesised.

Our ongoing synthesis of outcome evaluations will assess the effectiveness of interventions that integrate academic and health education in reducing substance use and violence outcomes. Our synthesis of theories of change is reported in detail elsewhere (currently under review). Briefly, the theory synthesis established that this category of intervention aims not only to integrate the teaching of health and academic education but also to bridge the relationships between staff and students so that affective bonds are strengthened, teachers serve more effectively as role models and students become more engaged in school. Many interventions also strive to generalise learning beyond the classroom to ensure that messages about health and academic education coming from the wider school and families are consistent with those taught in class and for reinforcement of knowledge and skills at multiple levels. The curricula and associated intervention components are further intended to normalise students’ positive behaviours to influence the development of social and emotional skills. These include, for example, self-management, empathy, communication and conflict resolution. Through these mechanisms, it is hypothesised that students will be less inclined to use substances, violence and aggression will decrease and academic performance will improve. To extend the usefulness of our review and to facilitate the design of future interventions that integrate academic and health education, a synthesis of factors affecting implementation of these interventions, documented in process evaluations, was undertaken.

Recent UK Medical Research Council guidance on process evaluations of complex interventions [11] stresses that these are useful in exploring what factors facilitate success. The process of designing more theoretically driven health improvement interventions has been hindered by the dominant paradigm within evidence syntheses, which is to focus on synthesising only quantitative studies answering questions about ‘what works’ [12]. Through synthesis of evidence on intervention processes, evaluators can develop hypotheses about the *contexts* within which interventions might be implemented and in which intervention mechanisms of action may produce intended outcomes, alongside findings about what works [13].

Although there are no existing syntheses of process data focused specifically on school-based interventions that integrate health and academic education, those examining the delivery of school-based health promotion interventions more generally can be found in the literature [14–21]. These identify constraining and facilitating factors operating at the school and class level, including the acceptability of the intervention to school staff and the adequacy of support for delivery. However, these factors are inconsistently defined and explored, making synthesis across studies challenging [22]. Theoretical frameworks also offer some suggestions as to what factors are likely to determine successful implementation. For example, May and Finch present normalisation process theory as a framework for understanding the sustainability of intervention implementation, suggesting a number of key factors: intervention coherence (people can make sense of a new practice), cognitive participation (people are willing to participate in a new practice), collective action (people are willing to take on the work required for the new practice) and reflexive monitoring (people are prepared to monitor the practice) [23].

However, no existing syntheses or theoretical frameworks have identified the factors that are likely to determine successful implementation of interventions integrating health and academic education in schools. This gap is likely because such integration is not seen as a focal component of the design of many interventions that use it, but rather, something that has emerged due to practical considerations. This may be one of the main reasons why these interventions remain under-developed. Therefore, we aimed to identify, appraise and synthesise available evidence from process evaluations to address the following research question: what characteristics of interventions, deliverers, participants and school contexts facilitate or limit successful implementation and receipt of interventions integrating health and academic interventions to prevent substance use and violence?

## Methods

### Review methods

Our overall review synthesised evidence on the theory of change, implementation and outcomes of interventions integrating health and academic interventions to prevent substance use and violence. Full methods are reported in a protocol included as a web appendix. The review followed PRISMA guidelines [24]. This paper reports on the synthesis of evidence on implementation. To be included in this synthesis, studies evaluated interventions delivered in classroom settings within mainstream public or private schools in regular school hours and integrating academic and health education to prevent substance use or violence among general populations of students aged 4–18 years. Included studies reported on the planning, delivery, receipt or causal pathways of interventions using quantitative and/or qualitative data. In October and November 2015, we consulted experts in the field of health education and social-emotional learning in schools to obtain their suggestions for possible included interventions or individual studies. From 18 November to 22 December 2015, we searched 19 health, social science and education databases. Searching of 32 websites and reference lists of relevant studies for further references followed between 12 and 23 January 2016. After carrying out the sample screen of 100 references to ensure more than 90% agreement, four reviewers independently screened the complete list of all possible included records on title and abstract. The full text of all records retained after this process were read in full by two reviewers to generate a final list of included studies that could answer at least one of our research questions (see Additional file 1 for further details).

### Data extraction and quality appraisal

We extracted data using a modified version of an existing tool [25] including items on study location; intervention/components, development and delivery; timing of delivery and evaluation; provider characteristics; target population; sampling and sample characteristics; data collection and analysis; and findings relevant to our review including verbatim quotes, author descriptions and interpretations of the findings. After piloting and refinement, two reviewers working independently extracted data from study reports and then met to agree on coding.

The reliability and usefulness of process evaluations was assessed by two reviewers using a standard tool for process studies—which has been widely applied in systematic reviews and informed by principles of qualitative research—[26] including the following six criteria: whether the sampling strategy was indicated; whether data collection methods were indicated—including any statements around increasing rigour of data collection; the degree of data analysis—including any statements around efforts made to improve reliability of findings and reduce bias; the extent to which the study findings were grounded in the data; the extent to which the study privileged the perspectives of intervention participants; and the breadth and depth of findings. Studies were assigned two types of ‘weight of evidence’ based on the reliability or trustworthiness of the findings and the usefulness of the findings for shedding light on factors relating to the research questions. Study reliability was judged as high when steps were taken to ensure rigour in at least four criteria, as medium when addressing only three and low when addressing two or fewer. To achieve a rating of ‘high’ usefulness, studies needed to be judged to have privileged the perspectives of intervention participants and to present findings that achieve both breadth and depth. Studies that were rated as ‘medium’ usefulness only partially met this criterion, and ‘low’ rated studies were judged to have sufficient but limited findings. Quality was used to determine the qualitative weight given to findings in our synthesis, with none of the themes represented solely by studies judged as low on both dimensions.

### Process evaluation data synthesis

Process evaluations commonly report qualitative, quantitative or mixed results. We anticipated that the quantitative data presented in included studies would address diverse questions and would therefore be too heterogeneous to meta-analyse statistically. Instead, textual reports of quantitative results were subject to thematic synthesis [27–29] after first checking that they were consistent with the quantitative data presented in the study reports. Studies were first read and re-read by two reviewers. The two reviewers then carried out line-by-line coding of process data in NVivo 11, developing inductive codes from these process data. Coding focused on textual reports which included verbatim qualitative data excerpts and author interpretations of these. Summaries of quantitative results were also coded in this manner after first checking that they were consistent with the quantitative data presented in the studies.

Each reviewer developed an emerging coding structure of hierarchically arranged codes applied in the course of the analysis. The two reviewers then compared their coding to agree on a common structure that formed the basis for the synthesis. As the overall analysis was developed, the reviewers referred to tables summarising the methodological quality of each study to ensure the synthesis reflected study quality.

## Results

### Search results

**Fig. 1:**
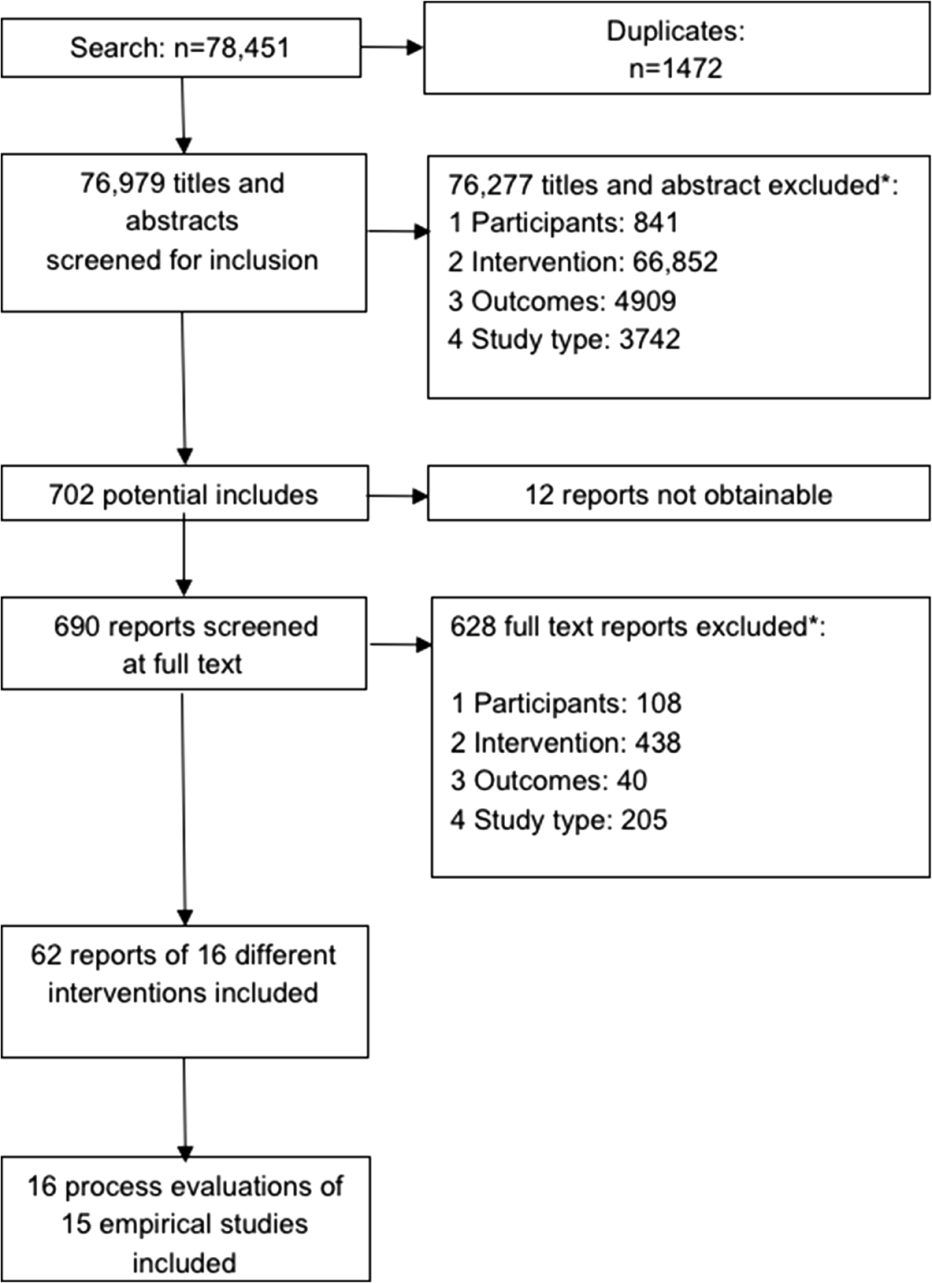
Flow of studies in the review

As per Fig. 1, after removing duplicates, 76,971 references were identified from the search (Additional file 2). From these, we included 16 relevant process evaluation reports that answered our research question on characteristics affecting implementation. These 16 reports presented data on15 empirical studies. One report (Hanson [30]) presented data on two separate studies. Two studies were each reported via two linked studies (Beets 2007 and 2008 [31, 32]; and Rothwell and Segrott and Segrott et al [33, 34]). There were 12 interventions reported on within these papers. A summary of all included studies of process and interventions is given in Table 1.

**Table 1:** Summary of interventions reporting on process

Intervention name	Description of intervention	Location	Targeted grade of participants	Process data collected on	Report
Reading Writing, Respect and Resolution (4Rs)	A literacy-based social-emotional learning curriculum for elementary school students. There are two components: (1) a seven-unit, 21–35 lesson literacy-based curriculum in conflict resolution and social-emotional learning for children in primary school (to grade five); and (2) intensive professional development for teachers.	USA	Kindergarten to grade 5	Fidelity and acceptability	Sung [39]
DRACON	This intervention uses drama to develop cognitive understanding of conflict and bullying and to empower students to manage their own conflict, both personally and within the broader school community.	Australia	Primary and secondary school students	Implementation, mechanisms of change, acceptability and context	O’Toole [37]
English classes (no name)	Teachers were trained and, working in pairs in the summer, they developed integrated health/English material, with a specific emphasis on the prevention of drug and alcohol use.	USA	Grades 8 and 9	Fidelity, acceptability, quality and mechanisms of change	Holcomb and Denk [43]
Hashish and Marijuana	The goal of the curriculum is to develop scientific knowledge of hashish and marijuana and to strengthen students’ problem-solving and decision-making skills through both didactic and participatory learning approaches.	Israel	Upper secondary school	Implementation	Zoller and Weiss [40]
Infused-Life Skills Training (I-LST)	A substance abuse prevention and competency curriculum that focuses on social and psychological protective factors affecting substance use. It is integrated into the existing subject curriculum by the classroom teachers.	USA	Middle/junior high school	Fidelity, quality, dose and acceptability	Bechtel et al. [42]
Kids, Adults Together (KAT)	The intervention aims to reduce drinking and antisocial behaviours in young people through a classroom curriculum, a parent evening and follow-up family activities.	UK	Grades 5 and 6	Acceptability and satisfaction	Rothwell and Segrott [33]
				Fidelity, reach and mechanisms of change	Segrott et al. [44]
Peaceful Panels	Throughout art classes, students participated in anti-bullying lessons (from the Second Step programme for eighth grade students on empathy and communication in handling a grievance) and comic-making lessons. They then prepared artwork to demonstrate their understanding of how to resolve conflict.	USA	Grades 8 and 9	Acceptability and satisfaction	Wales [45]
Positive Action	Positive Action is a social-emotional and character development intervention aimed at encouraging positive behaviours through positive thoughts and actions. Lessons cover six units: self-concept; positive actions for mind and body; positive social-emotional actions; managing oneself; being honest with oneself; and continually improving oneself.	USA	Kindergarten to grade 12	Coverage and acceptability	Beets [31, 32]
				Acceptability and satisfaction	Beets [32]
		USA		Implementation, fidelity, dosage and quality	Malloy et al. [41]
Promoting Alternative Thinking Strategies (PATHS)	An intervention to reduce conflict by improving students’ social-emotional and thinking skills through a curriculum, the establishment of a positive classroom environment and generalised positive social norms throughout the school environment.	USA	Kindergarten to grade 5	Quality, coverage (dose) and context	Ransford et al. [38]
Roots of Empathy	An intervention that brings a visiting baby and their parent into a classroom as a springboard for learning empathy. Students learn messages of social inclusion, respect, how to build consensus, how to contribute to a safe and caring classroom and develop emotional literacy.	Australia	Grades 1–9	Implementation, mechanisms of change and acceptability	Cain and Carnellor [34]
		Canada		Implementation and context	Hanson [30]
		UK			
Steps to Respect	This is an anti-bullying intervention with both school-wide and classroom components. The School-wide components create new disciplinary policies for bullying and improve monitoring of and intervention in bullying. Classroom curricula positive social norms and improve social–emotional skills for better engagement with bullying.	USA	Grades 3–6	Fidelity, context and acceptability	Low et al. [36]
The Gatehouse Project	Through teaching a curriculum and establishing a school-wide adolescent health team, Gatehouse aims to: build a sense of security and trust in students; enhance skills and opportunities for good communication; and build a sense of positive regard through participation in school life.	Australia	Grade 8	Coverage, quality and mechanisms of change	Bond et al. [35]

### Characteristics and quality of process evaluations

Of the 15 empirical studies, eight were conducted in the USA, three in Australia, two in the UK, one in Canada and one in Israel. Of the 12 interventions these studies summarised, four took place in primary or elementary schools, five in high or secondary schools and three in both (Table 1). Quality assessment is detailed in Table 2. Study reliability and usefulness varied. Only five reports were judged highly reliable and trustworthy, and five reports provided insights of ‘high’ value in answering our research questions. Six and five reports were respectively judged of ‘medium’ and ‘low’ reliability and trustworthiness.

**Table 2:** Quality appraisal of included studies of process

Intervention name	Site	Methods included steps to minimise bias in			Findings			Overall rating	
		Sampling methods	Data collection	Data analysis	Supported by data	Have breadth and depth	Privilege young people’s perspectives	Overall reliability and trustworthiness	Overall usefulness answering our research questions
4Rs (Reading Writing, Respect and Resolution)	New York, USA [39]	No Purposeful sampling but of only high-performing classrooms	Yes Different instruments piloted and used; findings triangulated	Yes Author verified data through ‘reflexive conversations’ and member-checking	Yes Clear results followed methods	Yes Very comprehensive data collected from a number of classrooms	No	Medium Selection of only high performers limits transferability of findings	High Detailed information about implementation provided
DRACON	Brisbane, Queensland and New South Wales, Australia [37]	No No detail provided	No No detail provided on increasing rigour	No No details were provided	No No quotations present to support qualitative data and no links to questionnaire data	No Good breadth of findings, but limited depth	Yes	Low Limited data on methods and links to results	Low Limited detail on implementation
English classes (no name)	Houston, USA [43]	No No detail provided	No No detail provided	No No detail provided	Yes Survey results followed clearly; qualitative results presented without supporting quotations	Yes Mixed methods enabled exploration of both breadth and depth	No	Low Limited detail on the rigour of methods used	Medium One of the few studies in which integration was core to the study’s design and some good detail around implementation is provided
Hashish and Marijuana	Haifa, Israel [40]	No No detail provided	No No detail provided	No No detail provided	No Scant data were provided, and it was unclear how these were produced	No Minimal findings reported	Yes	Low Poor reporting of methods and minimal results	Low Lack of detail in findings restricted the use of this study
Infused-Life Skills Training	PA, USA [42]	No No detail provided	Yes Multiple methods and instruments used; findings triangulated	No No detail provided	No No primary data provided, only authors’ accounts of the data	Yes Different aspects of implementation explored from students, teachers and administrators	No	Low Poor reporting of methods and minimal results	Medium Paper provides interesting insights and is the only one to compare with non-integrated curriculum implementation, but detail on methods is lacking
Kids, Adults Together (KAT)	Southeast Wales, UK [33]	No No detail provided	Yes Multiple methods used at different data points to ensure comprehensive perspectives	Yes Comparative coding used to refine analytical framework	Yes Clear results followed methods	No Good depth around acceptability, limited detail on other aspects of implementation	Yes	Medium Insufficient detail to determine possible bias introduced in sampling, but data collection and analysis seem appropriate	Low Nothing about the integration of academic and health curricula in findings
	Southeast Wales, UK [44]	No No detail provided	Yes Comprehensive qualitative data was collected	Yes Data were triangulated; constant comparison of data was done; and authors increased validity of instruments	Yes Although actual quotations and results from process evaluation were limited	Yes Data were collected on many aspects of implementation	No	Medium A lack of data on methods makes reliability impossible to ascertain	Medium This study has interesting findings but would be better to see them grounded in primary data
Peaceful Panels	Athens, USA [45]	No Convenience sample drawn from the author’s classroom	Yes A range of methods used to collect data and an independent peer audited the author’s methods	Yes Author employed reflexivity, debriefs with peers, and member-checking to increase robustness	Yes Clear results followed methods	Yes Considerable detail on a number of implementation factors reported	Yes	Medium Convenience sampling and (opinion of the study team) less-robust than possible analyses may limit trustworthiness	Medium Detailed information about implementation processes, but limited information about influencing factors
Positive Action	Hawaii [31]	Yes Sampling of schools was random, and there was an attempt to reach a census of all participating students	Yes Validated tools that collected data around a variety of measures of implantation were used	Yes Data were analysed using statistically appropriate methods	Yes Clear results followed methods	Yes Various features of implementation were explored in detail. These were generated from a large sample of diverse students. No qualitative data, however	Yes	High Methods were appropriate, efforts were made to increase rigour and the findings and interpretations lead clearly from the methods used	High This paper gives good information about important aspects of implementation
	Hawaii [32]	No Census of teachers attempted without success and no explanation provided	Yes High reliability of tools used	Yes Analysis were appropriate, and data were entirely quantitative	Yes Clear results followed methods	Yes Good range of process measures covered in considerable depth	No	High Study was well-conducted and statistically robust	High Useful discussion of key implementation factors including the perspectives of implementers
	Chicago [41]	Yes Relevant sampling criteria used with a very high response rate	Yes Multiple data sources used and triangulated	Yes Analytical approach was appropriate and robust	Yes Clear results followed methods	Yes Multiple data sources provided information about many aspects of implementation, in detail, with description of relationships between these	No	Medium Although methods were robust, there was no qualitative data to answer the more useful ‘why’ questions, particularly behind the relationships between implementation factors	High Good data provided around multiple aspects of implementation
Promoting Alternative Thinking Strategies	PA, USA [38]	Yes Sufficient detail provided; high (85%) response rate	Yes Alpha-reliability coefficients acceptable and provided; other measures of validity lacking	Yes Data analyses were appropriate	Yes Clear results follow methods	No Study limited to teachers’ psychological factors	No	High This is a well-conducted study	Medium Although methodologically sound, comprehensive results are lacking
Roots of Empathy	Western Australia [34]	No No detail provided	No No detail provided	No No detail provided	Yes Clear results followed methods	No Breadth around implementation from a teacher perspective, but little depth	No	Low A lack of methodological detail make trustworthiness questionable	Medium Useful data on some aspects of implementation provided, but lacking methodological rigour
	Western Canada and the Isle of Man, UK [30]	Yes Participants were from an ongoing RCT	Yes Reliability of instruments was good	Yes Data were merged to increase study power	Yes Constructs were well-defined and studied. Slight bias to Canadian results	No Lack of qualitative data	No	High This is a methodologically rigorous study	Medium Focus on teacher characteristics and implementation is valuable, but qualitative findings are limited
Steps to Respect	CA, USA [36]	Yes Participants are from an ongoing RCT; high response rate	Yes Questionnaire had high face validity and reliability	Yes Data analysis were appropriate	Yes Although qualitative exploration was lacking	No Concepts explored were limited	No	High This was a methodologically sound study	Medium Useful data, but qualitative findings are limited
The Gatehouse Project	Victoria, Australia [35]	No No detail provided on how participants were selected	Yes Multiple methods used to collect data at multiple points in the year	No No detail provided	Yes Clear results followed methods	Yes Multiple aspects of implementation were explored from multiple stakeholder perspectives	No	Medium More detail on methodological rigour would be required to make a fair assessment of robustness	High Very useful data provided around implementation characteristics

### Thematic synthesis of process evaluations

Five overarching thematic areas emerged, with one or more sub-themes related to implementation. These areas are support from senior school staff, teachers’ immediate working environment, teacher attitudes towards intervention characteristics, student attitudes towards intervention characteristics and parental support. These themes and their sub-themes are described below.

#### Support from senior school staff

Support from school managers and other senior staff, including administrators, was cited as a key driver of successful intervention implementation by eight authors (reporting on seven interventions) [30, 34, 32, 35–39], and two sub-themes were identified in the data.

##### A positive, supportive school climate that aligns with intervention goals was a facilitator

A supportive school climate is not only one in which school and intervention ethos overlap, but also one in which school managers are invested and consistently active in the intervention. In the case of the latter, provision of mentoring and coaching to teachers involved in intervention curricula and committing dedicated time and resources to the curricula was important. Consequently, teachers were more likely to feel a sense of support and connectedness to the school.

Although of medium reliability, findings from the Gatehouse Project process evaluation were deemed highly useful.

*Ongoing practical support from leadership has been acknowledged as important for mainstreaming the promotion of emotional well-being through promoting greater connections between learning, classroom practices, and student well-being. [One teacher recalls], ‘The support of my principal has to come number one…getting the time on the timetable, setting up a team, [that] can’t happen unless you’ve got someone in administration that thinks it’s a great idea.’* [35]*, p.378*

Furthermore, a sense of connectedness to the school meant that teachers felt aligned with the school’s decision to engage with the intervention in question, which helped to encourage teachers’ beliefs in and acceptance of their responsibility to teach the respective curriculum.

From one of the three highly reliable and useful studies of the Positive Action intervention in the USA, Beets et al. reported that:

*school leadership should develop a culture that encourages a shared and collective vision among staff and administration, is supportive of new innovations, and is aligned with the core values and concepts a given program is promoting … Perceptions of school climate were directly related to the beliefs teachers held about prevention/[social-emotional learning] and the attitudes teachers had towards [Positive Action].* [34]*, pp. 272–73*

##### A political and administrative environment that is amenable to an integrated curriculum is necessary

Support from ‘higher-up’ in terms of intervention alignment with political priorities, leading to dedicated policies and funding to facilitate an intervention’s implementation, was important for its success.

Despite being judged a lower quality study within our appraisal, implementers of Roots of Empathy in Western Australia noted that:

*Because of the financial support of [the Department of Education and Training] and its coordination of training, the program was successfully implemented. It is essential, however, that there is a strong policy and resourcing commitment to effectively sustain [the program].* [34]*, p.68*

Together, the factors identified in the two sub-themes were regarded as promoting greater implementation fidelity of integrated academic and health curricula.

#### Teachers’ immediate working environment

Teachers’ perceptions of their teaching environment as one that would be amenable to the intervention increased their own motivation for intervention delivery, with direct impact on implementation. Three sub-themes on this subject emerged from 10 studies of nine interventions [34, 40, 41, 35, 37–39, 42–44].

##### Teachers working collaboratively and learning from one another was a facilitator

For example, within the Positive Action intervention, [31, 32, 41] successful implementation was associated with teacher perceptions of their schools having an innovative culture and strong relationships between teaching staff. Authors suggested that these findings were due to schools with a capacity for innovation being perceived as more open to change and to new approaches, such as those championed by new interventions, which gave teachers more freedom to explore new programmatic areas [32, 41]. Strong relationships between teachers in Positive Action and other interventions were linked to a sense of mutual support and connectedness that teachers felt would help them to optimise intervention delivery [35, 42, 43].

Despite its lower quality rating in our appraisal, Bechtel et al.’s evaluation of the Infused-Life Skills Training intervention in the USA raised a number of useful insights including reports that:

*the first year participating teachers were especially helpful with recruiting and supporting new teachers in the program … they informally shared their experience with their fellow teachers, increasing interest and awareness of the program. They also gave examples of their lesson plans and discussed the importance of coaching and behavioral rehearsal in helping students master the life skills.* [42]*, p. 224*

##### Teachers feeling well-prepared to deliver the curriculum was essential

This sub-theme was raised more than any other (across six different interventions) and related to teachers feeling properly prepared and supported to deliver the curriculum [34, 37–39, 42, 44]. The consistency with which this issue recurred suggests this is essential to successful intervention delivery. This sub-theme was linked to the first theme of supportive schools as, often, much of this feeling of preparedness and confidence in delivery among teachers was instilled through support from management and other senior staff. More practically, the availability of intervention resources such as an easy-to-follow curriculum, adequate training and pre-prepared materials was highlighted as being very useful to teachers.

Within the Infused-Life Skills Training intervention:

*teachers reported that the training was critical in adequately preparing them to integrate [life skills] components into their curriculum. They indicated that the training was especially effective in their development and implementation of infused lessons, and that the step-by-step process and manual were valuable in guiding the development of their lesson plans.* [42]*, p. 224*

##### Teacher workloads and burnout is a barrier that should be overcome with administrative supports

Five studies identified teacher workload and/or burnout as a barrier to intervention implementation [37–39, 42, 43]. This may be partly addressed via school management support as above.

Limited methodological detail was provided by Holcomb and Denk in their study of English Classes, although many important aspects of implementation were explored. For example, they highlighted that:

*research [to implement integrated curricula] consumed additional time in the teachers’ already busy schedules and required teachers to ‘learn’ some of the materials before presenting them to their students. Teachers’ lack of time or access to information, in some cases, may have limited the amount of health content applied to individual lessons. Thus, interdisciplinary lessons sometimes were not as detailed as they could have been.* [43]*, p. S-39.*

This sub-theme links clearly to the one above, as teacher preparation for an additional curriculum responsibility contributed to burnout. With adequate training and administrative support—or collaboration with other teachers—teachers experienced less burnout and were more likely to implement the intervention successfully.

Within Ransford et al.’s high-quality study of Promoting Alternative Thinking Strategies:

*teachers who perceived their school administration as more supportive reported higher implementation quality, and positive perceptions of training and coaching were associated with higher levels of implementation dosage and quality. Teachers who reported the highest levels of burnout and the most negative perceptions of curriculum supports reported the lowest levels of implementation dosage and quality.* [38]*, p. 510*

#### Teacher attitudes towards intervention characteristics

Linked to teachers’ views on how supportive the school climate was for the implementation of these interventions, a key theme in several reports [32, 34, 35, 37, 40, 41] concerned the acceptability to teachers of the interventions themselves. This sub-theme was emphasised across studies as a factor enabling successful implementation to a greater extent than the acceptability of the intervention to students (see below), likely because teachers were typically the primary deliverers of the interventions.

##### Teacher belief in their responsibility to teach and own the integrated curriculum was a facilitator

Teacher uptake of interventions’ objectives was found to be linked to their attitudes towards the curriculum, their beliefs in their responsibility to teach social and emotional curricula and a sense of ownership of the integrated curriculum.

For example, Beets et al. reported from an evaluation of the Positive Action intervention in American primary schools that:

*teacher beliefs regarding their responsibility to teach [social-emotional learning] concepts were significantly…related to their attitudes towards Positive Action…[which] were positively related to the amount of the Positive Action curriculum delivered…and the amount of the curriculum delivered was positively related to material utilization in both the classroom…and school-wide.* [32]*, p. 217*

##### Positive teacher attitude towards and belief in the potential of the integrated curriculum was a facilitator

Teacher perceptions of the role of social and emotional learning—which was a part of the curriculum in all but two interventions included in the review overall—influenced their internalisation and subsequent role-modelling of the behaviours promoted within the curriculum [32, 34, 35, 38, 39, 41].

Emphasised in the Roots of Empathy intervention:

*all participants were committed to the importance of [social and emotional learning] in their teaching…they considered [it] essential to the academic learning that underpinned the teaching philosophy of all participants. The pedagogical understandings in the…program were consistent with each participant’s philosophy of learning and teaching.* [34]*, p. 63*

Conversely, teachers’ initial scepticism to new interventions or their feeling that these were a disruption to learning was barriers to implementation. Although the methods of this evaluation were poorly reported, the drama-based DRACON intervention in Australia experienced this barrier and the process evaluation explored this.

*A few [teachers] start with stronger reservations or resistance [to the programme], and some of these have chosen to withdraw from the project. These reservations are usually expressed as: not trusting drama to achieve its purpose, sometimes because it is perceived to potentially disrupt an orderly classroom, or to be too time-consuming in a full syllabus.* [37]*, p. 279*

##### Teachers’ freedom to be innovative and have flexibility within the curriculum was a strength

Curricula that were perceived by teachers to be adaptable to their classroom settings were generally implemented to a greater extent [40, 41, 43]. Some curricula were designed to be flexible, allowing teachers to adapt components of the intervention in line with the goals of their classroom and the topical interests of students.

One example of this type of flexible curriculum was found in the English Classes intervention in secondary Israeli schools reported by Holcomb and Denk:

*teachers reported that the program’s greatest strengths were its flexibility, its infusion of new material into their classrooms, and its interest to students … Autonomy allowed by the program was a significant strength noted by all the teachers, not only for the convenience it provided, but for the respect it displayed for their professionalism.* [43]*, p. S-39*

#### Student attitudes towards intervention characteristics

##### Students’ positive perception of the integrated curriculum was a facilitator

The acceptability of the intervention to students was reported as facilitating implementation, particularly where students saw the curriculum’s messages as relevant [31, 33, 40, 43–45].

Holcomb and Denk suggested that, ‘it was generally believed that the high level of interest among students was generated by the relevance of the health topics.’ [43], p. S-39

##### Students’ pre-existing attitudes aligning with intervention ideals was a facilitator

Not unlike teachers’ views about social-emotional learning, students’ pre-existing views of intervention messaging, if positive, were helpful in implementation. In Low et al.’s high-quality study of the Steps to Respect intervention in the USA, ‘significant positive associations with students’ engagement in the [Steps to Respect] lessons were found for classroom average levels of student support [of the programme], [and pre-existing] student attitudes against bullying, student climate and school connectedness.’ [36], p. 171.

##### Integrative interventions involving activities were regarded positively by students

Acceptability was greater where the learning activities that the interventions required were perceived to be relevant to students and fun to learn. For example, Wales et al. reported from an evaluation of the Peaceful Panels intervention in secondary schools in the United States that:

*although the students were not unanimous in positive feelings about the program, the great majority of them stated that they enjoyed it and that they felt that it helped them understand violence prevention … The students’ positive feelings implied that students enjoyed learning through comics and it is possible that this was this helped them retain what they learned.* [45]*, p. 143*

Students were particularly positive where a health/academic integration intervention encouraged teachers to focus on topics that were judged more relevant to students than traditional academic content or to use more participative learning methods than would usually be the case. An example from Bechtel et al. suggests that:

*students responded with interest and enthusiasm to the infused approach, liked the integration of substance abuse prevention into other subject areas, and were more engaged and eager to participate in class. Moreover, their students especially liked the facilitative classroom environment and the hands-on approaches of behavioral rehearsal and role playing.* [42]*, p. 224*

#### Parental support

##### A lack of parental participation and positive role-modelling of intervention concepts was a barrier

Parental involvement was in some cases a direct component of the intervention [33, 44] and thus a part of intervention implementation fidelity. Indirectly, parental involvement through reinforcement and role-modelling of curriculum messaging was sometimes part of the processes through which the intervention was hypothesised to work [33, 39, 44]. The role of parents could therefore be positive or negative and more often was indicated by authors as a barrier. For example, Sung reported in her account of implementation of the 4Rs intervention in primary schools in the USA, which was rated as highly useful, that:

*[an implementing teacher] … viewed inconsistency between the way students are taught at school and at home as an impediment. For example, whereas she taught children to ‘talk things out’ without using violence in a conflict, some parents encouraged their children to use violence as means of solving social conflicts at school.* [39]*, p. 100*

## Discussion

### Summary of key findings

Although factors that influenced implementation varied widely depending on the intervention, several—often linked—themes did emerge from our synthesis, namely around the necessity of senior management support, having a positive teaching environment, positive pre-existing teacher and student attitudes towards integrated health and academic interventions and favourable opinions about the autonomy and innovation that the interventions enabled, and parental support of interventions.

It is worth noting that many of the themes above relate to factors affecting implementation which might apply generally to school-based health promotion and social and emotional learning interventions. Here, we aim to draw out what our synthesis suggests about factors affecting the implementation of our specific category of interventions which integrate health and academic education. First, this category of intervention particularly benefits from consistent cross-school support from administrators and colleagues in integrating health across the curriculum. Strong networks, continuous training and shared understanding about the overall aims of integration take time to build and effort to sustain. Thus, ongoing support from administrators, both practically and in terms of morale, is crucial.

Second, interventions need to be flexible and locally adaptable if they are to mesh with the existing teaching environment and curriculum. Third, interventions that integrate academic and health education are innovative and challenging and so require teachers and staff to believe in, and commit to, integration as a longer term aim to improve students’ health and social and emotional learning. Such support appears to be promoted both because teachers value the scope they provide for local adaptation and professional autonomy and because students value the chance to learn using methods that are more participative and topics that appear more relevant to students’ lives than in standalone academic subjects.

To our knowledge, there are no existing reviews of interventions that integrate academic and health education. However, reviews of related interventions can help in contextualising our findings. In their review of health promotion interventions in schools, Chilton et al. [16] similarly noted that school (and teacher) cultural norms concerning substance use affected the extent to which interventions addressing this were successfully implemented. Staff investment overall was critical, including support from administrators. Likewise, Pearson et al. echoed the importance of engaging staff and suggested that, ‘implementation hinges on negotiation and programme delivery and the acceptability (or otherwise) of the programme to those who deliver it.’ (p. 17) They further commented on the importance of deliverers’ enthusiasm for the intervention and the need to root it in their perceived responsibility for its success [18]. Bonell et al.’s review of process evaluations of interventions aiming to increase the healthiness of school environments reported on the importance of a health intervention’s alignment with school ethos as a predictor of its success, as well as the importance to good implementation of the broad participation of all staff and support from administrators [15]. Rimm-Kaufmann and Hulleman noted similar factors in a review of social and emotional learning interventions in primary schools, emphasising teachers’ enthusiasm for interventions as being pivoted on their overall culture of education on these subjects and of these skills. Coupled with school-wide support and ongoing mentoring from higher-level staff, a supporting ethos enhanced teachers’ commitment to interventions and was thus crucial to their success [21].

Indeed, our review provides evidence that teachers’ perceptions of their school’s teaching culture was a key determinant of successful implementation, something which did not emerge as a key theme in the other reviews cited above. This factor may reflect the importance of genuine integration between health and mainstream academic elements when delivering this particular category of intervention [46].

Considering May and Finch’s normalisation theory [23] again as a framework to understand factors affecting the potential sustainability of these interventions, the roles of the teacher in understanding/internalising key components of the curriculum and enthusiastically taking responsibility for the intervention were facilitators of these interventions. Likewise, collective action through whole-school engagement and administrator support were also notable facilitators. There was no mention of reflexive monitoring in reports of process. However, reporting on these individual elements was inconsistent across interventions. Therefore, while some interventions like Positive Action, 4Rs, the Gatehouse Project and Roots of Empathy, which reported positively on these factors, seem conducive to sustainability, the potential sustainability of other interventions remains questionable.

### Implications for research and policy

Our findings suggest that integrative interventions, while attractive as ways to deliver some health, social and emotional learning in the context of school systems overwhelmingly focused on educational attainment, are not a panacea, as their implementation poses particular challenges. Proper integration requires that teachers believe in the interventions and have the time and resources to reflect and build-in a seamless integration, that interventions have enough flexibility to be applied effectively in diverse contexts and that the baseline teaching culture of a school is conducive to this type of intervention. This category of interventions will not flourish in instances where staff are demoralised and change jobs frequently, where they are sceptical about integrating health into their lessons or where managerial and collegial support for this challenging work of integration is perceived an issue.

Unfortunately, moderating factors—for example, the effects of gender, socioeconomic status, and so forth—were not examined by the authors of the studies we have included in this review, so they could not be included in our analysis. From other studies and reviews specific to interventions that emphasised social-emotional learning (which represents the majority of interventions included in our review), identified moderating factors include universal versus targeted interventions, the influence of the overall risk level of a school, the quality of schools’ interactions with students, students’ family environments and differential impact on boys versus girls and younger versus older age groups [14, 17, 19, 20]. It would be of interest to explore if and how such factors may play a role in the implementation and uptake of the integrated academic and health interventions, which may be of value in future research.

Many interventions in our broader systematic review prioritised reporting on outcomes over process (35 outcome evaluations were included, whereas only 16 relevant process evaluations could be found). This critique can be applied to the reporting of interventions more broadly. A review of implementation data by Michie et al. found that only 5–30% publications of experimental studies had detailed intervention descriptions at all [47]. This lack of detail presents issues when trying to produce replicable interventions. The paucity of intervention description is compounded further by a lack of explanation of the mechanisms by which interventions achieve outcomes and the contextual factors that may influence both implementation and outcomes [22, 48]. This gap has been noted by several authors, and across disciplines [22, 47–51].

Our current review confirms that process data were certainly less prioritised by authors, but further, that the quality of this reporting was poor. There was limited discussion of context in particular. Especially within complex public health interventions that aim to bring about behaviour change, both implementation and outcomes are inevitably influenced by context. Furthermore, understanding implementation through establishing how it was impacted by context, among other central implementation processes and factors, can prevent ‘type III’ errors—the wrongful attribution of intervention outcomes to an incorrectly implemented intervention [52].

A thorough exploration of implementation makes it possible to know whether an intervention has been implemented as intended and what considerations must be applied prior to its replication elsewhere. Realist approaches are helpful here, as they aim to thoughtfully test hypotheses around intervention implementation, noting explicitly the mechanisms that lead to outcomes and the contexts that influence these [48, 53]. Future research should therefore aim to incorporate a study of process alongside outcomes for a more robust understanding of intervention effectiveness.

Additionally, there is increasing recognition that evaluation needs to move away from accrediting specific interventions as effective/not and towards developing and refining theory of implementation. To do so, there needs to be sound documentation of how context influences intervention implementation alongside theories of change to detail how context interacts with intervention mechanisms to produce outcomes [48, 53]. Thus, process evaluations or other studies reporting on implementation must empirically examine how context influenced implementation to develop an implementation theory that is evidence-based [23]. Therefore, our synthesis of process data is a contribution to this literature, focused on a particular category of school-based health-promoting interventions. Simultaneously, however, it highlights a pressing need for further research into the processes of such interventions for their applicability to be fully appreciated.

### Method’s strengths and limitations

Our study involved a comprehensive search of available literature on the implementation of interventions that aim to integrate academic and health education to reduce substance use and/or violence. Given our robust searching methods, it is likely that we have captured, to the greatest extent possible, what is published about the implementation of these interventions. Our use of a standard tool to assess quality also added to the review’s rigour.

Although our analysis sought to employ a systematic and in-depth approach to synthesising the findings of process evaluation, it was somewhat limited by the paucity of relevant findings. While reporting on conventional, largely quantitative measures of implementation fidelity and acceptability, many studies failed to report on how implementation was affected by characteristics of interventions, deliverers, participants or school contexts and so contributed little to our synthesis.

## Conclusion

Several factors facilitating and inhibiting the implementation of interventions that integrate academic and health education to reduce substance use and/or violence are described here, providing tentative but insightful evidence of context-specific issues that may impact intervention success. However, overall, there is still a considerable gap in our understanding of how to achieve the successful implementation of these. With a view to promoting sustainability of these interventions within ever-changing socio-political and economic circumstances, more detail about context, moderating factors and facilitators and barriers at the individual, school-wide and community levels will be necessary. Our synthesis of effects of these interventions on violence and substance use is currently being completed for publication.

## Additional files

Additional file 1: PRISMA Checklist. (DOC 412 kb)

Additional file 2: Example search strategy. (DOCX 123 kb)
