# Supplementary material for: Interventions integrating health and academic interventions to prevent substance use and violence: a systematic review and synthesis of process evaluations
Source: Syst Rev. 2018 Dec 6;7:227. doi: 10.1186/s13643-018-0886-3 (PMC6284294; doi:10.1186/s13643-018-0886-3)
Supplement: Supplementary file 1 — PRISMA Checklist. (DOC 412 kb) [file 13643_2018_886_MOESM1_ESM.doc]

**How can interventions integrating health and academic education in schools help prevent substance misuse and violence, and reduce health inequalities among young people? Systematic review and evidence synthesis**

**Background**

This review will synthesise evidence on school-based curriculum interventions which integrate health and academic education to prevent substance misuse and violence among young people, and reduce inequalities in these outcomes.

*Description of the problem*

The proposed review focuses on substance use (alcohol consumption, smoking and drug use) and violence since these are important, inter-correlated outcomes which are addressed by interventions sharing common theories of change. Alcohol has been suggested to be the most harmful substance in the UK. Treating alcohol-related diseases costs the NHS in England an estimated £3.5 billion annually. The total annual societal costs of alcohol use in England are estimated at £21 billion. Alcohol related harms are strongly stratified by socioeconomic status (SES). Early initiation of alcohol use and excessive drinking are linked to later heavy drinking and alcohol-related harms and poor health. Alcohol use among young people is associated with truancy, exclusion, and poor attainment, as well as unsafe sexual behaviour, unintended pregnancies, youth offending, accidents/ injuries and violence. Preventing young people from taking up smoking is another key public health objective with 80,000 deaths due to smoking each year. In 2005-6, smoking cost the NHS £5.2 billion and wider costs amounted to £96 billion. Of smokers, 40% start in secondary school and early initiation is associated with heavier and more enduring smoking and greater mortality. Smoking among young people is a major source of health inequalities. Among UK 15-16 years olds 25% have used cannabis and 9% have used other illicit drugs. Early initiation and frequent use of ‘soft’ drugs may be a potential pathway to more problematic drug use in later life. Drugs such as cannabis and ecstasy are associated with increased risk of mental health problems, particularly among frequent users. Young people’s drug use is also associated with accidental injury, self-harm, suicide and other ‘problem’ behaviours. The proposed review’s other primary outcome is violence. The prevalence, harms and costs of violence among young people mean that addressing this is a public health priority. One UK study found that 10% of young people aged 11-12 reported carrying a weapon and 8% admitted attacking someone with intent to hurt them seriously. By age 15-16, 24% of students report that they have carried a weapon and 19% reported attacking someone with the intention to hurt them seriously. There are also links between aggression and anti-social behaviours in youth and violent crime in adulthood. As well as leading to further health inequalities, the economic costs to society of youth aggression, bullying and violence are high. For example, the total cost of crime attributable to conduct problems in childhood has been estimated at about £60 billion a year in England and Wales.

Many schools are reducing provision of personal, social and health education (PSHE) lessons which address these health issues because: PSHE is not a statutory subject (the government recently rejected advice that it should be); schools increasingly focus on narrow attainment targets; and school inspectors no longer report on schools’ promotion of student health and personal development.

*Description of the intervention*

Systematic reviews suggest that school curriculum-based health interventions

can reduce alcohol consumption, smoking, drug use and violence, but these are increasingly difficult to deliver within PSHE. In this context, many schools deliver health education in other subjects, integrating it with academic learning. Even without the marginalisation of PSHE, this approach may be more effective because: it could allow for larger doses; it may be less prone to student resistance and prevention fatigue; and it may enable synergy and reinforcements between sessions provided in different subjects. However, existing interventions of this sort have not been informed by existing theory or evidence. The UK can learn from evidence being generated in other countries. For example, the “4Rs” (**Reading, Writing, Respect & Resolution) programme** aims to integrate learning of social and emotional skills with literacy skills for children in US elementary schools. An RCT reported significant reductions in aggression and improved academic attainment.

In terms of theory of change (see appendix 1), such interventions may either incorporate health education into other mainstream school subjects or aim for health education lessons to include teaching of academic as well as health knowledge and skills. Such interventions could address substance use or violence by developing: social and emotional skills such as self-awareness, self-regulation, motivation, empathy and communication; healthier social support or norms among students; knowledge of the costs and consequences of substance use; media literacy skills to critique tobacco advertising; and modifying students’ social norms about substance use. This category of intervention will involve theories of change that are distinctive from conventional health education because such interventions aim to integrate health promotion into academic learning, and may aim to promote developmental cascades involving the interplay of cognitive and non-cognitive skills. Effects on substance use and violence are likely to be synergistic since each predisposes the other and has common risk factors.

*Rationale for the current study*

No systematic review has examined evidence concerning interventions integratinig health and academic education. The reviews cited above, some of which are now quite old, focused on school-based interventions but the interventions included are overwhelmingly those delivered in PSHE and international equivalents. Some of these reviews do include some interventions integrating health and academic education (described below under “Size of available literature”) but they omit important studies and do not analyse or draw conclusions about the effects of this specific category of intervention. Furthermore these reviews have not synthesised evidence on intervention theories of change or process evaluations and so cannot provide information about the feasibility and acceptability of interventions, or their transferability to the UK.

These are important gaps because of the marginalisation of PSHE in England, the potential advantages of interventions integrating health and academic education and the distinctive approaches and theories of change of this category of interventions. There is thus a good rationale for a new systematic review focused on this category of interventions. This review focuses on substance use (alcohol, smoking and drug use) and violence because: our scoping searches and logic model suggest that these interventions have most potential in reducing risk of these behaviours; substance use and violence are closely intertwined; and the theories of change underlying interventions addressing these outcomes appear to be similar.

**Research aim**

To search systematically for, appraise the quality of, and synthesise evidence to address the following research questions:

RQ1. What types of curriculum interventions integrating health and academic education in schools addressing substance use and violence have been evaluated?

RQ2. What theories of change inform these interventions and what do these suggest about their potential mechanisms and effects?

RQ3. What characteristics of interventions, deliverers, participants and school contexts facilitate or limit successful implementation and receipt of such interventions, and what are the implications of these for delivery in the UK?

RQ4. How effective are such interventions in reducing alcohol consumption, smoking, drug use and violence, and increasing attainment, when compared to usual treatment, no treatment, or other interventions, and does this vary according to students’ socio-demographic characteristics?

RQ5. What characteristics of interventions, deliverers, school contexts and students appear to moderate or are necessary and sufficient for the effectiveness of such interventions?

**Research objectives**

(1) To conduct electronic and other searches by December 2015.

(2) To screen found references and reports for inclusion in the review by February 2016.

(3) To extract data from, and assess the quality of, included studies by May 2016.

(4) To develop a typology of interventions and synthesise theories of change and process evaluations by September 2016.

(5) To consult with policy/practice and youth stakeholders on the typology and theory/process synthesis to inform amendments and plans for synthesis of outcome data by October 2016.

(6) To synthesise outcome evaluation data and undertake meta-regression and/or qualitative comparative analyses by December 2016.

(7) To draw on these syntheses to draft a report addressing our research questions by February 2017.

(8) To consult with policy/practice and youth stakeholders on the draft report to inform amendments and dissemination by March 2017.

(9) To submit the final report to NIHR by May 2017.

**Research design overview**

Our proposal is for a multi-method systematic review of the theories of change, process and outcomes of school-based curriculum interventions integrating health and academic education among students age 4-18 years addressing substance use or violence. The review will follow existing general criteria for the good conduct and reporting of systematic reviews (e.g. Centre for Reviews and Dissemination; Preferred Reporting Items for Systematic Reviews and Meta-Analyses). The review protocol will be registered with NIHR and with PROSPERO International Prospective Register of Systematic Review (http://www.crd.york.ac.uk/Prospero/).

**Size of available literature**

On 16 June 2014, we conducted a preliminary search in PubMed using the search string given in appendix 2. This was a limited search in that: (i) it searched only medical journals when it is likely that relevant studies are published in journals in the fields of education, psychology, sociology, criminology and public/social policy, as well as in sources other than journals; (ii) we used a limited search string; and (iii) it is not possible to use adjacency terms within PubMed. Our search identified 2,462 references, which were screened on title and abstract then full report. Of these, six reports (reporting on five distinct studies) met our inclusion criteria. In addition, we already knew of eight reports (from two studies), only one of which overlapped with the reports located via our PubMed search

Furthermore, the existing systematic reviews cited earlier between them include seven studies relevant to our proposed review, none of which was found in our own preliminary search. This lack of overlap suggests a substantial literature to be found with a more systematic search. Importantly, none of the existing systematic reviews analysed interventions integrating health and academic education as a specific category in narrative synthesis or meta-regression and drew no conclusions about their specific effects. As requested by the funding board we give full details here. Foxcroft and Tsertsvadze’s (2011) review of alcohol prevention identified two studies relevant to our proposed review. Thomas et al’s (2013) review of smoking prevention included three relevant reports (from two distinct studies). Thomas et al’s review found but excluded two studies relevant to our review (which were found in our own preliminary search). One was excluded because its outcome did not meet Thomas et al’s inclusion criteria, and another because at the time it was an ongoing study with no available data. Faggiano et al’s (2008) review of drug prevention included one study relevant to our review. Farrington and Ttofy’s (2010) review of bullying interventions identified two studies relevant to this proposal. Vreeman’s (2007) review of bullying interventions included no studies relevant to this proposal.

Summarising all relevant studies from all our various sources, ten) are outcome evaluations, and three studies are process evaluations. Four focus on multiple substances; three on smoking; one on drugs; and four on violence. In conclusion, the number of studies found and lack of overlap between the different sources suggests that our proposed systematic review with its more comprehensive search strategy is likely to identify a large but manageable number of studies. We found no economic evaluations and do not propose including these.

**Criteria for considering studies for this review:**

*Types of participant*

We will include studies conducted where a majority of participants are children and young people aged 4-18 years attending schools.

*Types of intervention*

We will include school-based health curriculum interventions integrating health and academic education targeting young people age 4-18. Academic education is defined as: education in specific academic subjects; literacy; numeracy; or study skills. It does not include: social conduct in the classroom; relationships with peers or staff; attitudes to education, school or teachers; or aspirations and life goals. Interventions may involve either incorporate health education into other, mainstream school subjects or aim for health education lessons to include academic education as well as teaching health knowledge and skills. Interventions may be delivered by teachers or other school staff such as teaching assistants, but may also be delivered by external providers, for example from the health, voluntary or youth service sectors. Our definition excludes interventions which: are delivered in mainstream subject lessons but do not aim to integrate health and academic education; train teachers in classroom management without student curriculum components; or are delivered exclusively outside of classrooms.

*Types of outcome*

We will include studies addressing one or more of the following primary review outcomes: smoking (e.g. salivary cotinine, carbon monoxide levels, self-reported use of cigarettes); alcohol use (e.g. self-reported alcohol consumption via questionnaires or diaries); legal or illegal drug use (e.g. self-reported drug use); and violence (self-reported violence perpetration - for example, carried weapon, got into a fight - and victimisation).

Informed by existing systematic reviews focused on substance use and violence among young people, outcome measures may draw on dichotomous or continuous variables, and self-report or observational data. They may use measures of frequency (monthly, weekly or daily), the number of episodes of use or an index constructed from multiple measures. Alcohol measures may examine alcohol consumption or problem drinking. Drug outcomes may examine drugs in general or specific illicit drugs, including drug convictions. Measures of violent and aggressive behaviour may examine the perpetration or victimization of physical violence including convictions for violent crime. Our Data Analysis section describes how we will combine measures.

Though not an inclusion criterion, we will assess academic attainment as a secondary outcome: e.g. student standardised academic test scores, IQ tests or other validated scales; school academic performance.

*Types of studies*

In order to address RQ 1 and 3, we will include studies reporting on process evaluations. This would include studies reporting on planning, delivery, receipt or causal pathways using quantitative and/or qualitative data. These studies may report exclusively on process evaluations or report process alongside outcome data. In order to address RQ 1 and 4, we will include studies reporting on outcome evaluations, using randomized controlled trials allocating schools, classes or individuals. Controls will be students, classes or schools allocated randomly to a control group in which no or usual school health and academic education is delivered, or to a control group including another ‘active’ intervention. In order to address RQ2 we will draw on included process and outcome evaluations as defined above which include descriptions of intervention theories of change or logic models. In order to address RQ5, we will draw on syntheses of all of the above study types.

**Search methods for the identification of studies**

In appendix 1, we provide the search string that we have used in a preliminary search in PubMed. As explained above, this was a limited search but it will inform the development of a more sophisticated search strategy maximising sensitivity as recommended by the Cochrane Handbook for Systematic Reviews of Interventions. Our search strategy will be informed by those used in previous systematic reviews focused on school interventions addressing alcohol, smoking, drug use and violence. The studies sought by this review are not likely to be reliably indexed in databases with controlled vocabularies. So we anticipate our searches involving a large number of free text terms. We will take the following three key concepts from the inclusion criteria to develop the search string: health education; integration with academic learning; and children and young people or schools. The combination of these concepts is sensitive enough to include all available studies regardless of study design. The three concepts will be linked by the Boolean operator “AND”. Our searches will involve different free text and controlled vocabulary terms for each of these two concepts linked by the Boolean operator “OR”. In our use of terms relating to health education, we will use a very broad array of terms to minimise the risk of publication bias. We will not restrict the searches by date, language or publication type.

*Electronic searches*

We will search the following databases from inception to present: ASSIA; Australian Educational Index; BiblioMap (Database of health promotion research); British Educational Index; Cochrane Central Register of Controlled Trials; Cochrane Database of Systematic Reviews; Database of Abstracts of Reviews of Effects; Database of Promoting Health Effectiveness Reviews; Econlit; ERIC; Health Technology Assessments; IBSS (International Bibliography of the Social Sciences); Medline; NHS Economic Evaluation Database; Proquest Dissertation Abstracts; PsycInfo; Social Policy and Practice including Child Data & Social Care Online; Social Science Citation Index/Web of Knowledge; and Trials Register of Promoting Health Interventions.

*Searching other resources*

We will carefully search reference lists from all studies that meet the inclusion criteria. We will hand-search journals that published included studies which we found only via reference checking and which are not indexed on databases we have searched (initially for the last 5 years and if these elicit >1 new included studies, for a further 5 years). We will search the following websites: the Campbell Library; Digital Education Research Archive; OpenGrey (System for Information on Grey Literature in Europe); Database of Educational Research; International Clinical Trials Registry Platform; Schools and Students Health Education Unit Archive. We will also search for relevant government reports and non-governmental organization publications via a Google search. We will contact subject experts to identify relevant ongoing or completed research. We will search all available clinical trials registers (e.g. clinicaltrials.gov) for relevant ongoing and unpublished trials.

**Data collection and assessment**

*Selection of studies*

Search results will be downloaded into EPPI-Reviewer 4. An inclusion criteria worksheet with guidance notes will be prepared and piloted by two reviewers screening the same 50 references. Where the two reviewers disagree, they will meet to discuss this and if possible reach a consensus. If the reviewers cannot reach consensus regarding inclusion of a specific article, judgement for selection will be referred to a third reviewer. If necessary, we will organise translation of papers published in languages in which we are not proficient. After piloting and any refinements, each reference will be screened on the basis of title and abstract for potential inclusion by one reviewer, using text-mining to prioritise screening the most relevant studies first. Full reports will be obtained for those references judged as meeting our inclusion criteria or where there is insufficient information from the title and abstract to judge inclusion. A second round of screening will then occur focused on full study reports to determine which studies are included in the review (see appendix 3: flow diagram). We will maintain a record of the selection process for all screened material.

*Data extraction and management*

Two reviewers will independently extract data from all studies meeting the inclusion criteria, using a piloted data extraction form with guidance developed for this review. Where the two authors disagree, they will meet to discuss this and if possible reach a consensus. If the reviewers cannot reach consensus regarding the particulars of data extraction for a specific study, judgement will be referred to a third reviewer.

Included studies will be described using the EPPI-Centre classification system for health promotion and public health research, supplemented by additional codes developed for this review. For all studies where relevant, we will extract information pertaining to: basic study details (individual and organizational participant characteristics, study location, timing and duration, research questions or hypotheses); study design and methods (design, allocation, blinding, sample size, control of confounding, accounting for data clustering, data collection, attrition, analysis); intervention characteristics (timing and duration, programme development, theoretical framework/logic model, content and activities, providers and details of any intervention offered to the control group); process evaluation of the intervention (feasibility, fidelity/quality, intensity, coverage/accessibility, acceptability, mechanism and context using an adapted version of an existing tool); outcome measures at follow-up(s) (reliability of measures, effect size both overall and where available by age, sex, socio-economic status and ethnic sub-group). The two reviewers will independently enter data from the data extraction forms into EPPI-Reviewer 4. If included studies are reported in languages that cannot be translated by the review team, a review author will complete the data extraction form in conjunction with a translator.

Published reports may be incomplete in a wide range of ways. For example: they may not report sufficient detail about their participants for our equity analysis; they may not present information on all the outcomes that were measured (possibly resulting in outcome reporting bias); they may not provide sufficient information about the intervention for accurate characterisation; and they may not report the necessary statistical information for the calculation of effect sizes. In all cases where there is a danger of missing data affecting our analysis, we will contact authors of papers wherever possible to request additional information. If authors are not traceable or sought information is unavailable from the authors within two months of contacting them, we will record that the study information is missing on the data extraction form, and this will be captured in our risk of bias assessment of the study.

*Assessment of quality and risk of bias*

We will assess the quality of theories of change using a modified version of the criteria developed in our ongoing NIHR-funded systematic review of positive youth development interventions, which for example assess the clarity with which constructs are defined and inter-related. We will assess the quality of the qualitative and quantitative elements of process evaluations using standard Critical Appraisal Skills Program and EPPI-Centre tools. These address the rigour of: sampling; data collection; data analysis; the extent to which the study findings are grounded in the data; whether the study privileges the perspectives of participants; the breadth of findings; and depth of findings. These are then used to assign studies to two categories of ‘weight of evidence’. First, reviewers will assign a weight (low, medium or high) to rate the reliability or trustworthiness of the findings (the extent to which the methods employed were rigorous/could minimise bias and error in the findings). Second, reviewers will assign an additional weight (low, medium, high) to rate the usefulness of the findings for shedding light on factors relating to the research questions. Guidance will be given to reviewers to help them reach an assessment on each criterion and the final weight of evidence. The two reviewers will then meet to compare their assessments, resolving any differences through discussion and, where necessary, by calling on a third reviewer.

For outcome evaluations, we will assess risk of bias within each included study using the tool outlined in the Cochrane Handbook for Systematic Reviews of Interventions. For each study, two reviewers will independently judge the likelihood of bias in seven domains: sequence generation; allocation concealment; blinding (of participants, personnel, or outcome assessors); incomplete outcome data; selective outcome reporting; and other sources of bias (e.g. recruitment bias in cluster-randomised studies); and intensity/type of comparator. Each study will subsequently be identified as ‘high risk’, ‘low risk’ or ‘unclear risk’ within each domain. In cases of disagreement, the reviewers will meet to seek consensus but where they cannot, we will refer judgement to a third reviewer.

We will assess reporting bias according to Sterne’s guidance. We will reduce the effect of reporting bias by focusing synthesis on studies rather than publications, avoiding duplicated data. Following the Cho statement on redundant publications, we will attempt to detect duplicate studies and, if multiple articles report on the same study, we will extract data only once. We will prevent location bias by searching across multiple databases. We will prevent language bias by not excluding any article based on language.

**Data analysis**

*RQ1 and 2: Thematic synthesis of intervention descriptions and process data*

Using thematic synthesis methods we will undertake a number of syntheses. Intervention descriptions (RQ1) and theories of change (RQ2) will first be analysed to develop a taxonomy of interventions integrating health and academic education. Syntheses of theories of change (RQ2) and process evaluations (RQ3) will be used to understand potential mechanisms of action. Syntheses of process evaluations (RQ3) will be used to understand: characteristics of interventions, participants and context acting as potential barriers and facilitators of implementation and receipt (RQ2); and an assessment of potential applicability to the UK. These syntheses will not be restricted to studies judged to be of high quality. Instead conclusions drawing on poorer quality evidence will be given less interpretive weight. First, the reviewers will prepare detailed evidence tables to describe: the methodological quality of each study; details of the intervention examined; study site/population; and full findings. Second, the two reviewers will undertake pilot analysis of two studies. The reviewers will read and re-read data contained within the evidence tables relating to the two high-quality studies, applying line-by-line codes to capture the content of the data. They will draft memos explaining these codes. Coding will begin with in-vivo codes which closely reflect the words used in findings sections. The reviewers will then group and organise codes, applying axial codes reflecting higher-order themes. The two reviewers will meet to compare and contrast their coding of these first two high-quality studies, developing an overall set of codes. Third, the two reviewers will go on to code the remaining studies drawing in the agreed set of codes but developing new in-vivo and axial codes as these arise from the analytical process, and again writing memos to explain these codes. At the end of this process, the two reviewers will meet to compare their sets of codes and memos. They will identify commonalities, differences of emphasis and contradictions with the aim of developing an overall analysis which draws on the strengths of the two sets of codes and which resolves any contradictions or inconsistencies, drawing on a third reviewer if necessary to achieve this. Through this process will be developed an explanatory framework to understand factors affecting implementation. Results will be presented to PPI stakeholders who will determine which interventions they think are applicable to the UK (see “Stakeholder collaboration” below).

*RQ4: Synthesis of outcome data*

We will first produce a narrative account of the effectiveness of these types of interventions. This narrative synthesis will be ordered by outcome then within this by age group, intervention type and follow-up time. Outcomes will be categorised into violence, smoking tobacco, drinking alcohol, using other drugs and academic attainment. Age will be categorised by the key-stage age-ranges used in the English educational system. Categorisation by intervention type will be informed by our prior thematic synthesis of intervention descriptions and theories of change through which we will have produced a taxonomy of interventions. This taxonomy may refer to: whether interventions incorporate health education into other, mainstream school subjects or aim for health education lessons to include teaching of academic as well as health knowledge and skills; lesson frequency; style of delivery; or other aspects of interventions which appear to be critical from our preliminary synthesis. We will describe study results in the ‘characteristics of included studies’ table, or enter the data into additional tables. We will then produce forest plots for each of our review outcomes, with separate plots for different outcomes and follow-up times, age groups and intervention types. Plots will include point estimates and standard errors for each study, such as risk ratios for dichotomous outcomes or standardised mean differences for continuous outcomes.

Once we know the number of studies and the extent of heterogeneity among the studies (as determined both by a Cochran’s *Q* test and inspection of the I^2^), we will make a decision whether to calculate pooled effect sizes. The results of statistical tests will be evaluated in accordance with the Cochrane handbook. If an indication of substantial heterogeneity is determined (e.g. study-level I² value greater than 50%) that cannot be explained through meta-regressions, then we will not produce a pooled estimate and will present only the narrative summary. If we consider that we have unexplained statistical heterogeneity in any of our study groupings, we will investigate this further using subgroup and sensitivity analyses. When studies are found to be statistically heterogeneous, we will use a random-effects model; otherwise we will use a fixed-effects model. When using the random-effects model, we will conduct a sensitivity check by using the fixed-effect model to reveal differences in results.

If we do produce pooled estimates, we will consider using a multilevel meta-analysis model to synthesise effect sizes. This is because outcome evaluations are likely to include multiple measures of conceptually related outcomes and multi-level meta-analysis improves on previous strategies for dealing with multiple relevant effect sizes per study, such as meta-analysing within studies or choosing one effect size, by including all relevant effect sizes but adjusting for inter-dependencies within studies. Unlike multivariate meta-analysis, it does not require the variance-covariance matrix of included effect sizes to be known. We will estimate separate models for substance use, violence and educational attainment outcomes. We will estimate separate models for substance use, violence and educational attainment outcomes, and for different age-ranges. We will examine substance use outcomes together in one analysis, as well as separated into smoking, alcohol, illicit drug use and any ‘omnibus’ measures of substance use. We will regard follow-up times of less than three months, three months to one year and more than one year post-intervention as different outcomes. We will run these models for interventions overall and where sufficient studies are found we will run separate models for different intervention categories and comparators. This categorisation will be informed by the taxonomy derived from our prior synthesis of intervention descriptions and theories of change. Finally, we will analyse the effectiveness of the subset of interventions identified by stakeholders as relevant to the UK context (see Stakeholder collaboration below). Where meta-analyses are performed, we will include pooled effect sizes in forest plots, with the individual study point estimates weighted by a function of their precision.

Prior to synthesis, we will check for correct analysis (where appropriate) by cluster and report values of: intra-cluster correlation coefficients (ICC), cluster size, data for all participants or effect estimates and standard errors. Where proper account has not been taken of data clustering, we will correct for this by inflating the standard error by the square root of the design effect.[63] Where ICCs are not reported, we will contact authors to request this information or impute one, based on values reported in other studies. Where imputation is necessary, we will undertake sensitivity analyses to assess the impact of a range of possible values. In other instances of missing data (such as missing population information), it may not be possible to include a study in a particular analysis if, for example, it is impossible to classify the population using our equity tool.

We will use the GRADE approach as described in the Cochrane Handbook for Systematic Reviews of Interventions to present the quality of evidence and ‘Summary of findings’ tables. The downgrading of the quality of a body of evidence for a specific outcome will be based on five factors: limitations of study; indirectness of evidence; inconsistency of results; precision of results; and publication bias. The GRADE approach specifies four levels of quality (high, moderate, low and very low). If sufficient studies are found, we will draw funnel plots to assess the presence of possible publication bias (trial effect versus standard error). While funnel plot asymmetry may indicate publication bias, this can be misleading with a small number of studies. We will discuss possible explanations for any asymmetry in the review in light of our number of included studies.

We will undertake a sensitivity analysis to explore whether the findings of the review are robust in light of the decisions made during the review process. We will also assess the impact of risk of bias in the included studies via restricting analyses to studies deemed to be at low risk of selection bias, performance bias and attrition bias. Where data allow, we will undertake additional exploratory meta-analyses to determine intervention effects on theorised intermediate outcomes (such as knowledge, skills, social norms) to examine the plausibility that these might mediate or otherwise precede behavioural effects. Such analyses will be informed by the synthesis of theories of change and process evaluation findings to avoid data-dredging. Where possible we will examine intervention effects by participant sub-groups (for example in terms of age, socioeconomic status, sex and ethnicity) and contexts (for example in terms of school-level deprivation) in order to examine potential impacts on health inequalities. This will draw on existing methods involving an ‘equity lens’ employing meta-analyses of subgroup effects from included studies and/or meta-regression drawing on studies with different participant or site characteristics to assess whether these moderate effects (see below for methods).

*RQ5: Meta-regression and qualitative comparative analysis*

If at least ten studies are found, we will employ meta-regression using Stata to investigate what factors moderate intervention effects in order to examine what characteristics of intervention, deliverers, contexts and students moderate effectiveness (RQ5). It may not be feasible to apply this method if we judge there are too many confounders or insufficient data, or if meta-regression is unable to account for interdependencies in complex interventions. Hence, we will complement meta-regression with qualitative comparative analysis, adapted for use in research synthesis to assess necessary and sufficient conditions for intervention effectiveness. As with our current review of positive youth development, the use of initial hypotheses derived from work addressing RQ 2 and 3 will protect us from ‘dredging’ the data for spurious statistically significant results. The required steps of ‘qualitatively anchoring’ outcomes in qualitative comparative analysis will ensure that changes in outcomes are meaningful and not simply statistical artefacts with little relevance for decision-making. We should stress that meta-regression and qualitative comparative analysis will be exploratory, hypothesis-building analyses since these will draw on observational rather than experimental comparisons.

**Stakeholder collaboration**

Prior engagement with the Ariel Trust, the PSHE Association and the Association for Young People's Health established the importance of this topic for English and UK schools. This prior engagement informed the contents of this proposal, for example the value of: synthesising process data to assess potential transferability to the UK; and collaborating with stakeholders to identify a sub-group of included studies most applicable to the UK to assess the effectiveness of this sub-group. We also consulted with students and teachers. Engagement with the ALPHA (Advice Leading to Public Health Action) young people’s group based at DECIPHer occurred on 29 October 2014. It involved 5 young women and 1 young man aged between 15 and 17 years. Discussions with this group confirmed that personal, social and health education (PSHE) was often not taught in a specific curriculum slot or, if it was, this received very little time. PSHE was often taught in student registration time or in specific ‘drop-down’ days, and this resulted in health education often being sporadic and rushed. Young people liked the idea of health education being integrated with learning in other subject lessons. It was felt that this could make health education more interesting and less ‘preachy’. The issue of the marginalisation of PSHE in schools has also come up in numerous other consultations with the ALPHA group when discussing the prevention of violence and substance use. Again, young people identify that it is increasingly difficult to deliver good interventions in PSHE sessions because of lack of time but that more thought and evidence is required before health education can easily be delivered in other parts of the school curriculum. We also consulted with 3 teachers at Chace School, Enfield (10 November 2014), and with two teachers and five male year 10 students at the Wren Academy, Barnet (10 September 2014). Consultation with teachers again suggested that in many schools PSHE was becoming more marginal in school timetables as a result of pressures on schools to maximise the time spent preparing students for testing and exams. These teachers were interested in the potential to teach students about health within academic lessons. They stressed that such work would need to be very well thought out to ensure a joining up of academic and health learning and so that academic learning was not compromised. They thought that violence and substance use might very well be addressed in maths, English, history and geography lessons. Students at Wren were also interested in the idea of teaching health within academic lessons. They stressed that such lessons would need to engage with the reality of students’ lives and the contexts within which violence and substance use occur. Finally, we discussed the potential review with the head-teacher of St Saviour’s Junior School in Westgate-on-Sea and three staff at Nightingale Primary School, Redbridge in December 2014. These staff suggested that PSHE was generally taught as a separate lesson in primary schools. Although in general the curriculum time available for PSHE is declining somewhat in primary schools, these declines are less dramatic than in secondary schools. Nonetheless several staff thought that there was particular potential for teaching health education integrated with academic learning among primary school students since academic progression and social development are so inter-linked among this age-group.

We will consult with policy/practice and youth stakeholders in the course of the project. We will convene an advisory group of the above policy/practice stakeholders, as well as a separate young people's advisory group run by ALPHA. Consultations with each of these will occur at two points: first, when we have synthesised evidence addressing intervention typologies and process evaluations, and second, when we are writing up the research. The first consultation will discuss the validity of our typology of intervention and synthesis of process evaluations, and will identify which interventions are deemed potentially feasible and acceptable in the UK. This will determine which interventions are included in our secondary analysis synthesising outcome data only for UK-applicable interventions. This innovative use of PPI has to our knowledge not been undertaken before in a systematic review. The second consultation will discuss the validity and usefulness of our syntheses and inform how research outputs are structured and disseminated. It will determine next steps in terms of knowledge transfer, replication studies and/or new intervention studies.

**Socioeconomic position and inequalities**

Socioeconomic status (SES) and health inequalities are central to the research. We will examine whether school-based curriculum interventions integrating health and academic education to prevent substance misuse and violence among young people lead to reductions in health inequalities in these outcomes. Where data allow, our review will use an ‘equity lens’, as described above. We will examine whether participant age, SES, sex and ethnicity, baseline risk behaviours and school-level deprivation moderate effects.

Dissemination and knowledge exchange

Our aim is to provide evidence on curriculum interventions integrating health and academic education in reducing children and young people’s substance use and violence. We will produce: a full technical report for NIHR; a briefing report for policy and practice audiences; and a concise young people’s report. The research will be launched at an event organised by the Association for Young People’s Health. Findings will be communicated to secondary schools via the UCLP and Welsh school health research networks. We will publish the research in open-access journals and via academic/policy conferences. We will undertake seminars with the UK and devolved national government departments of health and education, and national school inspectorates. We will use stakeholder and academic networks to support dissemination as well as existing web and social media platforms. There is great demand for evidence in this area. Schools are already integrating health with academic education because of the decreasing time for PSHE lessons. But such work is currently not evidence-based and may not be effective. Our review will provide evidence as to whether this approach is effective in reducing violence and substance use. It will also synthesise evidence on the sub-group of interventions which policy and school stakeholders identify as being most feasible in UK schools. Our review will identify which interventions appear to have the most potential to improve health outcomes and what theories of change, approaches and components such interventions employ. Depending on what we find, our review will either inform immediate knowledge transfer and scale-up of effective interventions or development of a research proposal either to the MRC Public Health Intervention Development stream or the NIHR Public Health Research Funding Board. The eventual aim would be for the scale-up of an intervention that schools can implement. We anticipate that an organisation such as the Ariel Trust or Mentor-UK would run a programme which schools or local authorities could purchase. It is also possible that our review might influence the contents of the national curriculum. Policy and practitioner support for this research is indicated by the active participation of Public Health England, Public Health Wales, the Association for Young People’s Health, the PSHE Association and numerous schools in developing the proposal.

**Research governance and ethics**

The principal investigator is responsible for the conduct and delivery of the work. The sponsor of the research is Steve Denton, Pro-Director of Strategy and Organisation at the Institute of Education. The co-applicants will form an investigator committee which will meet monthly throughout the project, overseeing its conduct. These meetings will be minuted to keep a record of tasks, deadlines and responsibilities. The research involves no human participants and draws solely on evidence already in the public realm, so IRAS approval is not required. Review and approval by the Institute of Education research ethics committee will be sought. The team will follow relevant guidelines and best practice. EPPI-Centre staff follow the Social Research Association’s (SRA) ethical guidelines and refer also to guidance recommended by the National Coordinating Centre for Public Engagement.

**Milestones**

(1) To conduct electronic and other searches by December 2015.

(2) To screen found references and reports for inclusion in the review by February 2016.

(3) To extract data from and assess the quality of included studies by May 2016.

(4) To develop a typology of interventions and synthesise theories of change and process evaluations by September 2016.

(5) To consult with policy/practice and youth stakeholders on the typology and theory/process syntheses to inform amendments and plans for synthesis of outcome data by October 2016.

(6) To synthesise outcome evaluation data and undertake meta-regression and/or qualitative comparative analyses by December 2016.

(7) To draw on these syntheses to draft a report addressing our research questions by February 2017.

(8) To consult with policy/practice and youth stakeholders on the draft report to inform amendments and dissemination by March 2017.

(9) To submit the final report to NIHR by May 2017.

**Expertise**

Professor Chris Bonell will direct the review overseeing all stages and components. He is experienced in leading systematic reviews and research on school-based interventions to promote health. Professor James Thomas is vastly experienced in systematic reviews and meta-analysis. He will oversee the methods used to synthesise statistical, narrative and qualitative data. Dr G.J. Melendez-Torres is an experienced systematic reviewer. He will contribute to leading the quantitative analyses. Dr Adam Fletcher and Professor Rona Campbell are both experienced in systematic review methods and research on school-based health interventions. They will contribute to developing and consulting on the typology of school-based curriculum interventions and synthesising process evaluations. They will also facilitate the involvement of DECIPHer’s youth advisory group, ALPHA, and the DECIPHer-led School Health Research Network in Wales. Professor Rob Anderson is experienced in systematic reviews of school-based health interventions. He will advise on our synthesis of process evaluations. Claire Stansfield is an experienced information scientist who will lead our electronic searches.

**References**

**Appendix 1**. Logic model of interventions delivered within mainstream subject lessons addressing substance use and violence

**Integration of health curricula into other school subjects**

Increase **feasibility / dose**

**Emotional and mental health**

e.g. wellbeing, quality of life, self harm, self esteem, depression, anxiety, strengths and difficulties

**Knowledge**

e.g. relating to costs and consequences of substance use

**Skills**

e.g. self-awareness, self-regulation, motivation, empathy and social skills

e.g. media literacy

Overcome **resistance to overt health education**

**Substance use**

e.g. salivary cotinine, carbon monoxide levels, self reported cigarettes, alcohol, and other legal or illegal drugs

**Synergy/ reinforcement** across lessons

**Social support**

**Social norms**

e.g. regarding substance use

**Appendix 2.** Exporatory search - interventions delivered within mainstream subject lessons addressing substance use and violence

PubMed 16/6/14

2462 hits

| Schools | AND | Curriculum (OR) |
| --- | --- | --- |
| schools [MeSH] |  | mainstream curricul*  mainstream subject*  mainstream lesson*  academic curricul*  academic subject*  academic lesson*  literacy  numeracy  math* lesson*  math* teach*  math* curricul*  history lesson*  history teach*  history curricul*  literature lesson*  literature teach*  literature curricul*  language lesson*  language teach*  language curricul*  geography lesson*  geography teach*  geography curricul*  science lesson*  science teach*  science curricul*  biology lesson*  biology teach*  biology curricul*  physics lesson*  physics teach*  physics curricul*  chemistry lesson*  chemistry teach*  chemistry curricul*  art lesson*  art teach*  art curricul*  humanities lesson*  humanities teach*  humanities curricul* |

**Appendix 3**. Flow diagram

Electronic searches

Other searches

References downloaded to Eppi Reviewer 4

Duplicates removed

Screening for inclusion

**RQ1.** What types of curriculum interventions delivered within mainstream subject lessons in schools addressing substance use and violence have been evaluated?

**RQ2.** What theories of change inform these interventions and what do these suggest about their potential mechanisms and effects?

**RQ3.** What characteristics of interventions, deliverers, participants and school contexts facilitate or limit successful implementation and receipt of such interventions, and what are the implications of these characteristics for delivery in the UK?

**RQ4.** How effective are such interventions in reducing alcohol consumption, smoking, drug use and violence, and increasing attainment, when compared to usual treatment, no treatment, or other interventions, and does this vary according to students’ socio-demographic characteristics?

**RQ5.** What characteristics of interventions, deliverers, school contexts and students appear to moderate/are necessary and sufficient for the effectiveness of such interventions?

Exclusions

Drawing on evidence from RQ1-RQ4
