# Supplementary material for: Interventions integrating health and academic interventions to prevent substance use and violence: a systematic review and synthesis of process evaluations
Source: Syst Rev. 2018 Dec 6;7:227. doi: 10.1186/s13643-018-0886-3 (PMC6284294; doi:10.1186/s13643-018-0886-3)
Supplement: Supplementary file 2 — Example search strategy. (DOCX 123 kb) [file 13643_2018_886_MOESM2_ESM.docx]

**Example search strategy**

This additional file provides an example of all of the string searches that were used for Psycinfo (OVID). It was searched on the 18 November 2015 and returned 22,451 references. Other database searches were based on this strategy.

1. ((substance? or drug? or drinking or alcohol* or solvent?) adj1 ("use" or abus* or misuse*)).ti,ab.

2. ((substance? or drug? or drinking or alcohol* or solvent?) adj1 (usage or intake or using or taking or behavio* or user?)).ti,ab.

3. (drinking adj1 (alcohol* or behavio*)).ti,ab.

4. Alcohol.ti,ab.

5. (smoke or smoking or tobacco or cigarette? or smoker? or cannabis or marijuana).ti,ab.

6. (aggression or aggressive or bully* or delinquen* or "conduct problem*" or "conduct disorder?" or "antisocial" or "anti social" or violence or violent or (volatile adj behavio*) or victimi* or hostile or hostility or perpetrat*).ti,ab.

7. (Externalising or externalizing).ti,ab.

8. emotion*.ti,ab.

9. PSHE.ti,ab.

10. ("Health literacy" or "health education" or "health promotion" or "preventive health" or "primary prevention" or "health information" or "promoting health" or "health promoting" or "health promotion" or "health maintenance").ti,ab.

11. "Public health".ti,ab.

12. ("wellbeing" or "well being").ti,ab.

13. "mental health".ti,ab.

14. 1 or 2 or 3 or 4 or 5 or 6 or 7 or 8 or 9 or 10 or 11 or 12 or 13

15. ((curric* or lesson? or classes or classroom? or subject? or intervention? or program* or education or initiative? or learn or learning or teach or teaching or outcome* or attainment or achievement or assessment or effect* or impact* or score? or scoring* or skill? or knowledge or competen* or performance) adj3 (Academic or academically or Scholastic or scholar* or Mainstream or "main stream")).ti,ab.

16. ((curric* or lesson? or classes or classroom? or subject? or learn or learning or teach or teaching or attainment or achievement or assessment or score? or scoring* or skill? or knowledge or competen* or performance) adj3 School?).ti,ab.

17. ((intervention? or program* or initiative? or effect* or impact* or education) adj1 School?).ti,ab.

18. (class adj1 (Academic or academically or Scholastic or scholar* or School? or Mainstream or "main stream")).ti,ab.

19. ((curric* or lesson? or classes or classroom? or subject? or education or learn or learning or teach or teaching or attainment or achievement or score? or scoring* or skill? or knowledge or competen*) adj3 (study or core or generic)).ti,ab.

20. (class adj1 (study or core or generic)).ti,ab.

21. ((curric* or lesson? or classes or classroom? or subject? or attainment or achievement or assessment or score? or scoring* or competenc* or performance) adj3 ((Education not ("patient education" or "continuing education")) or educational)).ti,ab.

22. (class adj1 ((Education not ("patient education" or "continuing education")) or educational)).ti,ab.

23. (outcome* adj1 (education or educational)).ti,ab.

24. ((curric* or lesson? or classroom? or classes or subject? or intervention? or program* or initiative? or education or teach* or outcome* or attainment or achievement or assessment or effect* or impact* or score? or scoring* or skill? or knowledge or competen* or performance) adj3 (learn or learning)).ti,ab.

25. (class adj1 (learn or learning)).ti,ab.

26. ((curric* or lesson? or classes or classroom or class or subject? or education or teach* or learning or teach or teaching or learn or attainment or achievement or assessment or score? or scoring* or skill? or knowledge or competen* or performance) adj3 (art or arts or math* or science? or humanities or chemistry or physics or language* or geography or (history not ("medical history" or "health history" or "familial history" or "family history")) or numeracy or (literacy not "health literacy") or grammar or grammer or reading or writing)).ti,ab.

27. (((curric* or lesson? or classroom or classes or subject? or skill?) adj3 literature) or "literature class").ti,ab.

28. ("Education reform" or "Instructional support" or "School reform" or "Classroom organi*" or (Commit* adj3 (school or education or learning)) or (Engag* adj3 (school or education or learning)) or "Character development" or "Whole school" or "School level" or "School wide" or schoolwide).ti,ab.

29. ((Comprehensive adj3 school) and (intervention? or program* or initiative? or outcome* or effect* or impact*)).ti,ab.

30. ((Integrat* or Combin* or Infuse or infused or infusion or sustainable) adj3 (curric* or lesson? or classes or classroom or syllabus or subject? or education or learn or learning or teach or teaching)).ti,ab.

31. (((Integrat* or Combin* or Infuse or infused or infusion or sustainable) adj3 (intervention* or program* or initiative*)) and school?).ti,ab.

32. ((school or education or core or generic or teaching or learning) adj3 syllabus).ti,ab.

33. 15 or 16 or 17 or 18 or 19 or 20 or 21 or 22 or 23 or 24 or 25 or 26 or 27 or 28 or 29 or 30 or 32

34. (child* or schoolchild* or youth* or "young people*" or "young person" or teen* or adolescen* or juvenile* or preadolescen* or boy? or girl?).ti,ab.

35. (curric* or lesson? or classes or classroom? or subject? or school? or syllabus or "junior high" or "senior high" or "junior education" or "elementary education" or "primary education").ti,ab.

36. 34 and 35

37. ("secondary school?" or "primary school?" or "comprehensive school?" or "school education" or "high school?" or "grammar school?" or "private school?" or "public school?" or "mainstream school*" or "compulsory education" or "statutory education" or "middle school?" or "junior school?" or "senior school?" or "primary education" or "secondary education" or "elementary school?" or "elementary education" or "mainstream education" or "compulsory school*" or "statutory school*" or "sixth form college?" or "post-16 education" or "junior high" or "senior high" or "reception class" or "post primary").ti,ab.

38. ((school? or junior? or elementary or senior? or primary or "sixth form" or grade) adj10 student?).ti,ab.

39. pupil?.ti,ab.

40. 36 or 37 or 38

41. (University or universities or freshmen or sophomore? or "higher education" or "tertiary education" or ((registrar* or workplace? or clinical or medical or nursing or nurse? or doctor? or continuing or adult? or patient?) adj1 (education or educating or profession* or student?)) or "professional education").ti.

42. 40 not 41

43. 14 and 33 and 42

44. "Elementary School Students"/ or "Intermediate School Students"/ or "Primary School Students"/ or "Middle School Students"/ or "High School Students"/ or "Junior High School Students"/ or "Kindergarten Students"/ or "High School Education"/ or "Middle School Education"/ or "Secondary Education"/ or "Junior High Schools"/ or "High Schools"/ or "Schools"/ or "Elementary Schools"/ or "Middle Schools"/

45. "Drug Abuse Prevention"/ or "Health Education"/ or "Drug Education"/ or "Health Promotion"/ or "Public Health"/ or "Health Promotion"/ or "Preventive Medicine"/ or Health behaviour/ or Harm reduction/ or Health literacy/ or exp Health screening/ or Primary Mental health prevention/ or Prevention/ or Public health/ or Lifestyle changes/ or Lifestyle/ or Health literacy/

46. "Tobacco Smoking"/ or "Smoking Cessation"/ or "Marijuana Usage"/ or "Drinking Behavior"/ or "Social Drinking"/ or "Binge Drinking"/ or "Underage Drinking"/ or "Alcohol Abuse"/ or "Alcohol Drinking Patterns"/ or "Alcohol Intoxication"/ or "Alcoholism"/ or "Heroin Addiction"/ or "Drug Addiction"/ or "Drug Dependency"/ or "Drug Usage"/ or "Inhalant Abuse"/ or "Drug Abuse"/ or "Glue Sniffing"/ or "Predelinquent Youth"/ or "Cyberbullying"/ or "School Violence"/ or "Teasing"/ or "Juvenile Delinquency"/ or "Physical Abuse"/ or "Verbal Abuse"/ or "Violence"/ or "Harassment"/ or "Antisocial Behavior"/ or "Bullying"/ or "Perpetrators"/ or "Threat"/ or "Victimization"/ or "Relational Aggression"/ or "Aggressive Behavior"/ or "Behavior Problems"/ or "Behavior Disorders"/ or "Conduct Disorder"/ or "Drug Education"/ or "Drug Abuse Prevention"/ or "Harm Reduction"/

47. emotions/ or emotional development/

48. emotional adjustment/ or emotional disturbances/ or emotional control/

49. mental health/ or primary mental health prevention/ or well being/

50. "Curriculum"/ or "Curriculum Based Assessment"/ or "Curriculum Development"/ or "School Learning"/ or "Classroom Environment"/ or "Academic Environment"/ or "Teacher Effectiveness"/ or "Teacher Effectiveness Evaluation"/ or "Educational Program Evaluation"/ or "Course Evaluation"/ or "learning environment"/

51. 14 or 45 or 46 or 47 or 48 or 49

52. 33 or 50

53. 42 or 44

54. 51 and 52 and 53
